# LINCS Data Portal 2.0: next generation access point for perturbation-response signatures

**DOI:** 10.1093/nar/gkz1023

**Published:** 2019-11-08

**Authors:** Vasileios Stathias, John Turner, Amar Koleti, Dusica Vidovic, Daniel Cooper, Mehdi Fazel-Najafabadi, Marcin Pilarczyk, Raymond Terryn, Caty Chung, Afoma Umeano, Daniel J B Clarke, Alexander Lachmann, John Erol Evangelista, Avi Ma’ayan, Mario Medvedovic, Stephan C Schürer

**Affiliations:** 1 Department of Molecular and Cellular Pharmacology, Miller School of Medicine, University of Miami, USA; 2 Center for Computational Science, University of Miami, USA; 3 BD2K-LINCS Data Coordination and Integration Center, USA; 4 Laboratory for Statistical Genomics and Systems Biology, Division of Biostatistics and Bioinformatics, Department of Environmental Health, University of Cincinnati College of Medicine, USA; 5 Sylvester Comprehensive Cancer Center, Miller School of Medicine, University of Miami, USA; 6 Department of Pharmacological Sciences, Mount Sinai Center for Bioinformatics, Icahn School of Medicine at Mount Sinai, New York, USA

## Abstract

The Library of Integrated Network-Based Cellular Signatures (LINCS) is an NIH Common Fund program with the goal of generating a large-scale and comprehensive catalogue of perturbation-response signatures by utilizing a diverse collection of perturbations across many model systems and assay types. The LINCS Data Portal (LDP) has been the primary access point for the compendium of LINCS data and has been widely utilized. Here, we report the first major update of LDP (http://lincsportal.ccs.miami.edu/signatures) with substantial changes in the data architecture and APIs, a completely redesigned user interface, and enhanced curated metadata annotations to support more advanced, intuitive and deeper querying, exploration and analysis capabilities. The cornerstone of this update has been the decision to reprocess all high-level LINCS datasets and make them accessible at the data point level enabling users to directly access and download any subset of signatures across the entire library independent from the originating source, project or assay. Access to the individual signatures also enables the newly implemented signature search functionality, which utilizes the iLINCS platform to identify conditions that mimic or reverse gene set queries. A newly designed query interface enables global metadata search with autosuggest across all annotations associated with perturbations, model systems, and signatures.

## INTRODUCTION

The Library of Integrated Network-Based Cellular Signatures (LINCS) (1) is an NIH Common Fund program with the goal of generating a large-scale and comprehensive catalogue of perturbation-response signatures by utilizing a diverse collection of perturbations (e.g. chemical, genetic, disease state), model systems (e.g. cell lines, differentiated cells, embryonic stem cells) and assay types (e.g. gene expression, protein expression, epigenetic modification, imaging). Currently at its Phase 2, LINCS consists of six Data and Signature Generation Centers (DSGCs) and one Data Coordination and Integration Center (DCIC) that together have produced over 400 datasets and over 50 analytical tools focusing on the deeper understanding of complex diseases and the development of novel and effective therapies.

Similarly to other large data generation efforts (2–6), the LINCS Data Portal (7) (LDP) offers a long-term central resource for the LINCS project and facilitates the accurate and uncomplicated sharing of all of its data. This has been achieved by adopting state-of-the-art big data infrastructure solutions, together with adhering to the well-established data management and stewardship guidelines of the FAIR guiding principles (Findable, Accessible, Interoperable, and Reusable) (8). Moreover, by following the systematically-developed LINCS Metadata Specifications (9) throughout the development of the LINCS Data Portal, we have ensured the highest integrity and quality of the stored data and metadata. Since its initial release, the LINCS Data Portal has been utilized by >18 568 unique users that downloaded >109 538 datasets belonging to 25 different assay types, thus, providing the scientific community with the most comprehensive and highly curated resource for perturbation-response signatures.

In this first major update of the portal (available at http://lincsportal.ccs.miami.edu/signatures), we are introducing substantial changes in both the data architecture and the user interface to enable a deeper exploration of the LINCS data, and support new integrative and analytical capabilities for both computational and non-computational researchers.

## NEW FEATURES AND FUNCTIONALITIES

### LINCS Data Portal 2.0

The LINCS Data Portal is the primary data access point for the LINCS Project and, since its initial launch, has supported the exploration and accessibility of one of the largest perturbation-response signature collections available. With the release of LINCS Data Portal 2.0 (LDP 2.0), we are introducing multiple major changes in both the backend and the frontend of the portal that provide researchers with new ways to browse and query the LINCS data and enable them to quickly and intuitively perform elaborate analytical tasks.

In this concerted effort to improve the user experience and re-affirm the LINCS commitment to FAIR principles, the cornerstone of this update has been the decision to reprocess all high-level LINCS datasets and make them accessible at the data point level. By making the individual LINCS signatures searchable and accessible, we eliminate the cumbersome step of users having to manually subset the LINCS dataset packages in order to locate data points that are relevant to their scientific questions, simultaneously improving the findability, accessibility, and re-usability of the LINCS data. Furthermore, users are offered the capability to quickly analyze selected LINCS signatures, using our expanding collection of external tools such as the iLINCS platform (http://www.ilincs.org).

**Figure 1.**
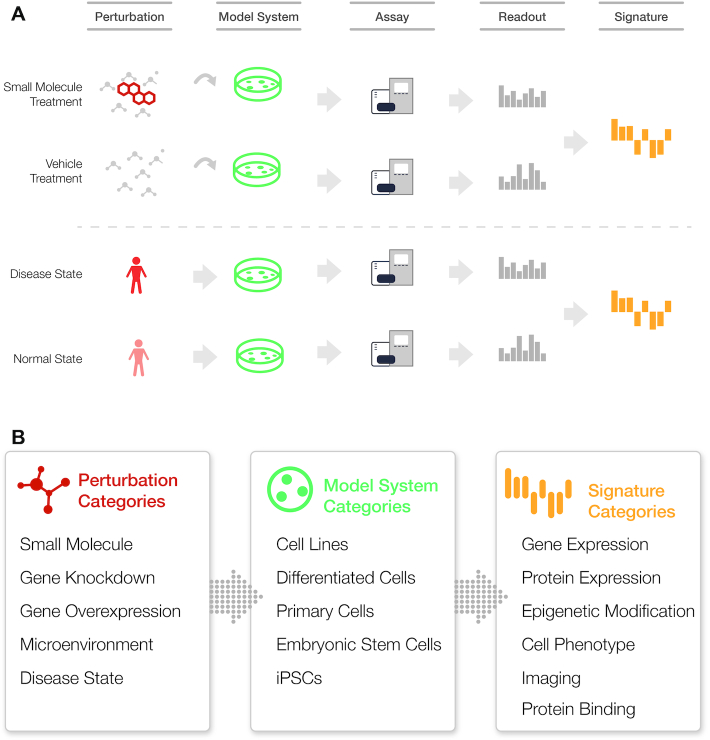
Illustration of generation and composition of Signatures. (**A**) Main components of a signature: perturbation, model system, and the readout generated in an assay. The final numeric signature is generated typically based on differential analysis of perturbed versus unperturbed states and suitable normalization and further processing (e.g. aggregation across replicates). (**B**) categories of perturbations, model systems, and signatures in LINCS.

Building on our previous metadata specifications efforts (9), we created an intuitive conceptual model for the LINCS signatures to accommodate for their unique annotation requirements. Using this model, a LINCS signature can be described by a combination of three key elements (Figure 1A): (i) the perturbation of the signature, (ii) the model system that was profiled to create the signature and (iii) the assay/readout of the signature.

Each of the above three key elements is then divided into subcategories that reflect the reagents and assay types that were used in the LINCS experiments (Figure 1B).

Our highly curated metadata annotations for signature perturbations, model systems, and readouts give users the ability to filter signatures using keywords that point to any of the above categories, thus, quickly and efficiently locate the exact LINCS signatures they are interested in exploring. Moreover, our completely redesigned UI makes the signature search tasks even more intuitive, as the above metadata categories are represented with unique and consistent UI elements throughout the portal.

Apart from our rich annotation efforts, we also aimed for a fast, reliable and scalable data infrastructure, in order to support the ever-growing number of signatures generated by the LINCS DSGCs. For this, we completely overhauled our initial data schema and created a hybrid architecture where the detailed metadata annotations of the signatures are stored in a relational database and the signature data themselves are stored in a NoSQL database. This hybrid architecture enables a fast searching across multiple metadata fields and facilitates the query, retrieval and comparison between the individual data values. Finally, the modular state of both the UI and the data infrastructure allow for future updates that will further enhance the capabilities of the portal.

In the sections below, we describe in detail the three key areas of the new LINCS Data Portal: (i) LINCS Signatures, (ii) New Website Functionalities and (iii) New Hybrid Data Storage Architecture.

### LINCS signatures

The most important change in LDP 2.0, was the transition from the LINCS dataset packages to the LINCS signatures. In the original version of LDP, the data produced by the LINCS DSGCs, were aggregated and organized in the form of dataset packages. A dataset package includes: (i) a readme file containing key annotations of the dataset (e.g. assay name, grant number, PI name, Center Name), (ii) the original data files as produced by the DSGCs, (iii) standardized metadata files for all key reagents used in the data files and (iv) mapping files that associate metadata files with the data files. Users were able to browse through the perturbations and model systems that were used in the LINCS datasets, select a LINCS dataset, and download it. This approach, however, created some extra challenges, since in every case, the user had to download the complete dataset, even when needing a small subset of its data points. Moreover, in cases where the queried model systems and perturbations were split across multiple datasets, the user needed to download each dataset separately and then filter and aggregate the correct data points.

In this new update, signatures are defined as changes in the assay readout of a perturbed sample (e.g. compound treatment, gene overexpression, gene knockdown, disease state) when compared to a control sample (e.g. vehicle treatment, scrambled controls, normal tissue) and can be calculated for all the dataset packages stored in the original LINCS portal. By transitioning to this signature model, we not only streamlined the above use cases, but we also are offering multiple new functionalities that will further enhance the integration and analytical capabilities of LDP. Using our deep metadata annotations, users can quickly search across all the LINCS data, select signatures that match their query and then proceed with the download and/or analysis using external tools (e.g. iLINCS).

### New website functionalities

The cornerstone of LDP 2.0 is the extensively redesigned User Interface that facilitates a global and integrative view of the LINCS data across all the different perturbations, model systems and assays of the LINCS project. To make the portal easy to use across both computational and experimental researchers, it was important to design a simple but informative web interface that would require a minimal learning curve for users. For this, all elements of the user interface were designed around our clear representation of a signature (perturbation + model system + assay). This simple conceptual model is consistent throughout the different pages of the portal and enables the users to intuitively search, locate, and download the data points of interest.

#### Global metadata search

**Figure 2.**
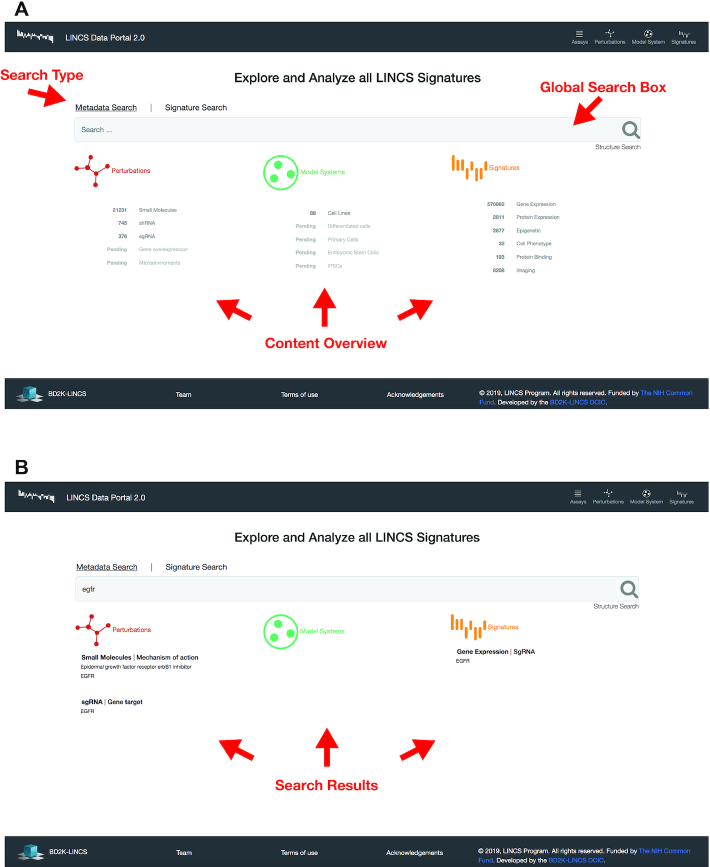
The LINCS Data Portal 2.0 homepage. (**A**) The LDP 2.0 UI facilitates simultaneous metadata search across three categories: perturbation, model system, and signature (readout) using the ‘Global Search Box’. In addition, the home page links to signature (similarity) search and chemical structure search as well as several catalogs to browse assays, perturbations, model systems and signatures (see Figure 3). (**B**) The results of the metadata search are displayed under the global search box and are grouped based on their categories (perturbations, model systems and signatures).

The home page of LDP 2.0 (Figure 2A) gives users a brief overview of the types of perturbations, model systems and assay readouts that constitute the LINCS signatures along with the ability to query across all the available signatures and metadata. One of the biggest challenges in designing a simple to use, function-rich search interface is the complexity of the LINCS data. Unlike other data portals, where the data points are annotated based on one dimension of metadata (e.g. based on tumor sample, patient, or cell line), a user of LDP 2.0 needs to be able to query across three separate categorical metadata dimensions (perturbations, model systems and readouts). Moreover, each of the metadata categories can be split into a large number of diverse subcategories. This can create the extra hurdle that depending on the context of the search term, the same search term can match multiple entities in multiple categories. For example, the search term *‘EGFR’* (Figure 2B) can refer to an sgRNA for EGFR, a compound that targets EGFR, a genetically modified cell line that constitutively overexpresses EGFR, or an assay that measures the mRNA expression of EGFR. Bringing all these different contexts under the same UI was a complicated endeavor which we solved by our unique home page UI.

Overall, the homepage is composed of two main elements (Figure 2B). A global search box and three text areas beneath, that directly represent our conceptual model of a signature. After a user enters a search term in the box, the three columns beneath get updated to reflect terms that match each respective annotation category. Matches of annotations related to a perturbation of a signature are shown in the perturbation column, matched terms related to metadata annotations of a signature's model system are shown in the model system column, and matches related to the readout of a signature are shown in the signature column. Importantly, the search terms can be matched to either a canonically accepted name (i.e. Ritonavir) or a synonymic name (i.e. Norvir).

#### Global signature search

In addition to querying signatures based on metadata annotations, all signature data values are queryable in LDP 2.0. For example, a user can search for signatures that upregulate a given list of genes, or that have a concordant profile with a preselected signature via the ‘Signature Search’ tab (Figure 2A). Overall, the ability to search using both the metadata keywords and data values empowers users with unprecedented capabilities to explore, analyze, and effectively utilize the comprehensive perturbation-response library produced by LINCS.

#### Perturbation, model system and assay pages

**Figure 3.**
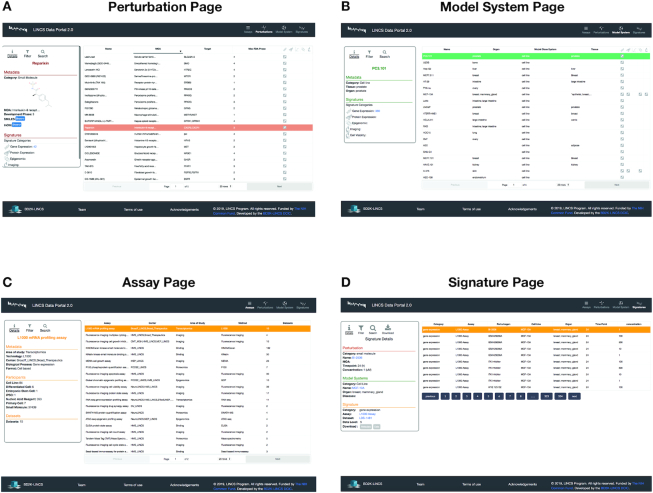
Screenshots of the catalog pages with an example detail panel for perturbations (**A**), model systems (**B**), assays (**C**) and signatures (**D**). The catalogs provide a list of records that match the search criteria or can be used to browse the content. The list can be further filtered based on several criteria that are category specific. The detail panel provides further information about the selected record, including metadata and annotations.

In order to adhere to our conceptual representation of a signature, we created individual pages that catalogue all the corresponding reagents and assays (Figure 3A-C). Through these pages, the user can browse and filter all the subcategories of the perturbation reagents (e.g. small molecules, sgRNAs, shRNAs, antibodies and disease states) and model system reagents (cell lines, iPSCs, differentiated cells, and primary cells) based on their extensive metadata annotations. Furthermore, users can explore all the 25 LINCS assays as well as key information, protocols and datasets associated with them, by using the dedicated assay pages (Figure 3C). Links to various external resources are also available through the individual reagent pages.

#### Signature pages

Another central feature of this update is the Signature Pages (Figure 3D). Here, all the LINCS signatures are listed and can be filtered by an assortment of criteria. When a user clicks on an individual signature, a details panel appears on the left side to offer key information regarding the perturbation, the model system, and the assay used. The modularity of this interface offers the capability to expand the left-side panel with future functions and analytical tools, according to the scientific community's feedback. Such actions can include: direct connections to other external tools such as iLINCS (http://www.ilincs.org) and L1000CDS2 (10), which offer on-the-fly statistical analysis such as Gene Set Enrichment Analysis (GSEA) (11) and signature similarity search.

#### Chemical structure search

LDP 2.0 includes data for over 20 000 small molecules, including approved drugs, compounds in clinical trials, and many tool-compounds and probes. Chemical structures of these compounds are stored in a database, and are searchable by substructure and similarity for the benefit of drug discovery researchers. The chemical structure search was implemented using ChemAxon Marvin JS (https://chemaxon.com/products/marvin-js), the structure search is executed using the API functionality by querying the JChem PostgreSQL Cartridge (https://chemaxon.com/products/jchem-engines).

#### Programmatic access (APIs)

The final considerably improved feature of LDP 2.0 is its rich and accessible application programming interface (API). The comprehensive API powers the frontend user interface and allows users to both find and access the data programmatically. It is essential to make LINCS data readily accessible by various types of users, both to allow for user preference as well as ensure adherence to the FAIR guidelines. The API is annotated using Swagger 2.0 JSON implementation, and all RESTful endpoints return structured JSON. Multiple endpoints allow searching content by any of its curated metadata annotations as well as requesting different slices of metadata associated with any of the subcategories of perturbations, model systems, and signature elements. The API also provides the chemical structure search functionalities including substructure similarity, and exact matches for all small molecules.

### New hybrid data storage architecture

**Figure 4.**
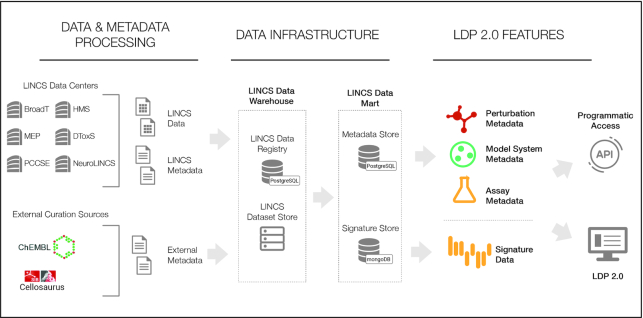
Illustration of the key stages of LINCS internal and external data and metadata processing from the data generating centers and data sources into the final standardized representation in LDP 2.0 accessible via API and UI. Via the LDP 2.0 data infrastructure, data and metadata are initially ingested from various sources into a data warehouse where they are standardized and integrated. From that warehouse, the data are loaded into an optimized hybrid data storage system consisting of a relational database and an object store to maximize performance and integrity for structured metadata and very large unstructured/diverse signature datasets (see text). Via an API and the optimized LDP 2.0 UI, all signature information are readily accessible in their final standardized description including perturbation-, model system-, and assay metadata and the signature numeric data and metadata (see text).

LDP 2.0 uses a hybrid architecture for data storage (Figure 4), combining a relational database and a document store to maximize overall performance while also ensuring the integrity of the data and metadata. Relational databases such as PostgreSQL excel at storing and searching structured data in a well-defined schema. While this comes at the expense of having to define the data structure ahead of time, it allows easy updating and indexing and very fast searches. Relational databases also enforce constraints based on the definition of primary and foreign keys and thus ensure the integrity of the data. Their downside is that indexing requires a relatively large amount of storage space and would be detrimental for large datasets that are inherently unstructured. NoSQL systems by contrast work extremely well at storing unstructured data, with most NoSQL systems having no defined schema. Such system, e.g. MongoDB, which is used in LDP 2.0, can be indexed to speed up certain searches, however, attempting to enforce a structured schema would be difficult. With the large variety of data and metadata produced by LINCS, a combination of these technologies represents a means to cleanly manage structured data such as small molecules, cells, genes, without compromising performance of searching very large sets of signature vectors, which are stored as sets of key value pairs. Furthermore, by leveraging the combination of data technologies alongside a properly implemented schema for data storage, we have improved on the LINCS data conformity to the FAIR guidelines.

Signature data produced in the LINCS project and made accessible via LDP 2.0 are a combination of different readouts from different assays and different instruments with varied normalization methods. Consequently, it is inherently unstructured. At the same time, however, it is unlikely to change once released by the data generators and submitted to the data portal. Their unstructured, varied nature and large size makes a relational system a poor choice for these data, as a different table would be needed for every type of submitted data, substantially increasing the required overhead. Conversely, a NoSQL database where a schema need not be defined and searches of individual readout values are unlikely to occur often, works well. Systems such as MongoDB used in LDP 2.0 enable aggregate searches across many different JSON documents thus easily allowing slicing of the data. Storage is not a major concern as scaling is simple and can be spread over multiple servers effortlessly.

Unlike the signature data, metadata such as perturbations or cell lines along with their various curated annotations, are more likely to be updated as new information or reference resources become available. However, metadata as a whole are small in size compared to the signature data. Importantly, metadata, in contrast to the signatures are highly structured and broken down into multiple classes of objects such as small molecules, genes, antibodies, proteins, cell lines, stem cells, assays, etc. These classes of objects are inherently linked to one another via the signatures that they relate to. The specific objects of the various categories have to be clearly and uniquely defined and support validation against external reference resources; they must be easily and specifically searchable, and it must be possible to update any of the information without risking to compromise data integrity. These requirements make a NoSQL system a poor choice. In contrast, a relational system, such as PostgreSQL used in LDP 2.0 perfectly supports all these requirements as it is designed to maintain the relationships between different classes of objects. The LDP 2.0 PostgreSQL database has tables for each class of object and defined relationships with strong restrictions preventing erroneous updates. Indexing enables very fast searching for nearly any attribute of the metadata without major slowdowns as the size of the tables expand.

The combination of these two technologies represents an efficient design to store and search large unstructured datasets as well as highly structured and uniquely identified metadata. Relationships are easily maintained with a unique ID between the two systems. It should be noted that our hybrid database design is not the first of its kind, as other systems such as Apache Druid and HadoopDB taking a similar approach of splitting the metadata and data.

## NEW DATA AND CURATED ANNOTATIONS

### Small molecule physicochemical and ADME properties

Physicochemical characteristics and basic ADME (absorption, distribution, metabolism, excretion) properties of small molecules and drugs, for example their molecular weight, aqueous solubility, lipophilicity, cell permeability, oral absorption, general protein binding, etc. influence how they perturb biological systems. The basic knowledge of these properties can inform early-stage structure modifications to obtain desired molecular profiles, improve experimental design, optimize dosing and treatment regimens, and determine best practices for administration routes in animal studies and ultimately reduce the probability of early failure in clinical trials. However, the prediction of physicochemical and ADME properties is largely unknown, unintuitive, or inaccessible to basic scientists. LDP 2.0 includes over 20,000 FDA approved drugs, chemical probes, and other small molecules at all phases of testing. The LDP small molecule landing pages feature prominently displayed physicochemical properties and color-coded property values to indicate favorable and unfavorable values. Property value calculations uniquely incorporate contributions for protonation states and tautomers significantly present at physiological conditions and using up-to-date calculation methods in Schrodinger QikProp (QikProp, version 3.5, Schrödinger, LLC, New York, NY, 2012) and ChemAxon Predictors (ChemAxon Marvin JS, version 19.20.0(rf0795bca7a2e), ChemAxon Ltd, Budapest, 2019). LDP provides a collection of commonly shown physicochemical properties in global leadlikeness and bioavailability properties and uniquely includes advanced ADME predictions for aqueous solubility, oral absorption, human serum albumin binding (khsa), blockage of HERG K+ channels (herg), blood and blood-brain barrier permeability, blood-brain partition coefficient for orally delivered drugs, skin permeability, and likelihood of metabolic reactions. Overall, the updated LINCS Data Portal 2.0 provides a broadened and more nuanced scope of molecular properties at physiological conditions to a broader and more diverse audience of researchers, further enabling interoperability and re-use of all LINCS data.

### Curated MOA annotations

As small molecules constitute the majority of the LINCS perturbation types, it was of high importance to enrich the perturbation-response signatures with valuable and highly curated metadata annotations. For this, we utilized widely used chemical repositories and extracted and curated mechanism of action and target annotations for all the small molecules that were used in LINCS.

Mechanism of action annotations were first downloaded from ChEMBL (12), CLUE (https://clue.io/), Drug Repurposing Hub (13) and Drugbank (14) using the available file transfer protocols (FTPs) and APIs from each data repository. In order to integrate between the different small molecule identifiers that each resource uses, we utilized our in-house small molecule standardization pipelines and mapped each external small molecule identifier to our LINCS Small Molecule (LSM) identifiers. Briefly, our small molecule standardization pipeline includes the removal of predefined salt and addend forms, neutralizes charges of deprotonated acid and protonated base forms, and generates a unique chemically reasonable tautomeric representation followed by SMILES canonicalization. Any exceptions detected by the pipeline are then manually reviewed and curated. The final step of the pipeline includes the mapping of the standardized structures, to PubChem compound identifiers (CIDs) via structure-based query of PubChem's Power User Gateway API.

### Curated target annotations

Molecular target data, primarily in the form of single proteins, were obtained from ChEMBL (12), in the form of ChEMBL target ID, Uniprot ID and protein name. In addition, MOA annotations were linked at the database level based on the procedures outlined above, and linked to specific protein targets based on information in ChEMBL and CLUE (https://clue.io/). The Uniprot/SwissProt (15) database API was utilized to obtain and verify information about gene source of the target as well as the target's organism, amino acid sequence, and disease association, if available. MOA targets and curated targets are searchable to quickly and intuitively identify small molecules with activity against a target of interest.

### Cell annotations

Canonical, immortalized cell lines utilized for LINCS projects were verified, curated, and annotated both through API/automated mechanisms and manual curation. Automated curation primarily consisted of matching cell line names to canonically given names in the Cellosaurus and Cell Line Ontology (CLO) (16,17) resources. This included obtaining synonymic names, source organism, clinical annotations (ethnicity, age, gender, etc., for human samples), and disease associations. As with small molecules, incoming cell line names are automatically recognized as standard or synonymic, and linkages were leveraged for proper naming in the final data structure. Disease associations were verified programmatically and manually through the Cellosaurus API, with the given disease checked against the reported disease in the API and flagged when differences were discovered. Manual curation acted as a vital step in this process, as many cell lines were registered with little supporting metadata and/or contained overlap in key aspects amongst canonically different lines. For example, the name PC-3 was given to two very distinct human cell lines in their initial isolation—one a prostate carcinoma cancer line (Cellosaurus ID CVCL_0035) and the other lung adenocarcinoma cancer line (Cellosaurus ID CVCL_S982). Cases like PC-3 exemplify the continued need for manual expert curation in metadata along with more extensive and thorough data sharing processes to provide high quality and trusted annotations for users to search and to improve overall FAIRness of data.

## FUTURE DIRECTIONS

With this major update of the LINCS Data Portal, we introduce a refined data portal that enables faster and more intuitive exploration of the diverse and complex data produced by the LINCS consortium. The design changes to the UI and the data model are instrumental in showcasing the value of LINCS signatures and in particular to provide integration across different signature types, for example, based on small molecule perturbations or cell lines, but also across different types of perturbations, such as a small molecule target and a gene knockdown. Importantly, the new portal makes LINCS data more accessible and searchable at the level of individual signatures. These updates will likely further increase the utilization of LINCS data across various research projects.

Through the new scalable data infrastructure and modular UI design, we are ensuring the longevity of the data portal and its positioning as a central analytical hub that will grow as more signatures, methodologies, and tools become available. Currently, LDP 2.0 integrates closely with iLINCS enabling signature-based search to identify perturbations that either mimic or reverse a reference signature. In future releases, we plan to integrate with other signature-based analytics tools, such as Enrichr (18) or SynergySeq (19) and additional sources of signatures including gene expression via Clue (https://clue.io/) and Creeds (20) or proteomics data in piLINCS (http://eh3.uc.edu/pilincs/). An exciting application of LINCS data is the discovery of molecular mechanisms and potential drug targets associated with cellular perturbations. To enable such research, we will further improve small molecule annotations from high-quality and curated resources such as BindingDB (21) and DrugCentral (5), and explore how we can incorporate high quality target predictions (22,23). Predictions for understudied proteins, such as those explored by the Illuminating the Druggable Genome (IDG) project (https://druggablegenome.net/) (24) provide starting points for the validation of new prospective drug targets.

Another future goal is to improve interoperability of different signature types, for example between the P100 phosphoproteomics data (25), KINOMEscan, and gene expression data by formalizing relationships between the different readouts, such as by utilizing the DTO (26). Finally, we also aim to include external signature types, such as standardized and validated Tox21 (27) reporter gene signatures that we have already processed for integration with LINCS signatures.
